# MCPIP: a key player in macrophage polarization

**DOI:** 10.18632/oncotarget.5451

**Published:** 2015-09-01

**Authors:** Pappachan Kolattukudy

**Affiliations:** Burnett School of Biomedical Science, College of Medicine, University of Central Florida, Orlando, Florida, USA

**Keywords:** Immunology and Microbiology Section, Immune response, Immunity

Metchinikoff's recognition that macrophages from infected animals had elevated bacterial killing ability 120 years ago laid the foundation for the concept of macrophage activation. Interferon γ (IFNγ) produced by lymphocytes was identified as the first factor that activated resting macrophages to enhance phagocytic activity and production of proinflammatory cytokines. This state became known as the classically activated state (M1). Later, IL-4 produced by Th2 cells was found to inhibit INFγ-induced respiratory burst and enhance the expression of MHC-II suggesting a special activation state of macrophages that became known as M2 state [1, 2]. M1 macrophages produce numerous proinflammatory mediators including TNFα, IL-1, IL-6, reactive oxygen and nitrogen species implying strong bactericidal and tumoricidal activities. M2 macrophages express their own characteristic molecules such as resirtin-like-a (Fizz1), Arginase 1 (Arg1), Chitinase 3-like (Ym1), IL-10 and Mrc1(CD206) that play a role in parasitic infestation, tumor progression, tissue remodeling, angiogenesis etc. It is recognized that M1 and M2 represent two extreme states of polarization but in reality activated states of macrophages represent a full spectrum with M1 and M2 being the two extremes.

Macrophage polarization state depends on their microenvironment that provides the cues for reversible states of activation. The plastic gene expression profile of macrophages is influenced by the type of stimulating agent in the microenvironment, its concentration and duration of exposure to the stimulant. The gene expressions plasticity is critically important for the adaptive response of macrophages as they migrate to different microenvironments in response to chemotactic agents and perform their functions [3]. The plethora of environmental cues that determine the macrophage activation state include cytokines, chemokines, hormones, TLR ligands, endogenous ligands such as integrin ligands, PPAR ligands etc. When the microenvironmental signals disappear macrophages revert back to the original state. Thus in vitro it can be demonstrated that when agents such as LPS that promotes M1 state or IL-4 that induce M2 state are removed the macrophages revert to the original state even though it is known that tissue specific signals may control the development of tissue specific macrophage phenotypes.

In recent years considerable progress has been made in our understanding of how the microenvironmental signals cause macrophage polarization. LPS and IFNα-induced M1 polarization and IL-4-induced M2 polarizations have been studied extensively. TLR activation by LPS leads to NFkB activation that regulate production of inflammatory cytokines characteristic of M1 macrophages. M2 polarization is involved in resolution of inflammation. Stimulation of IFNα receptor triggers JAKmediated tyrosine phosphorylation leading to dimerization of STAT-1 that binds to promoters of genes encoding NOS2, the MHC II transactivator and IL-12 leading to the production of the M1 markers.

**Figure 1:**
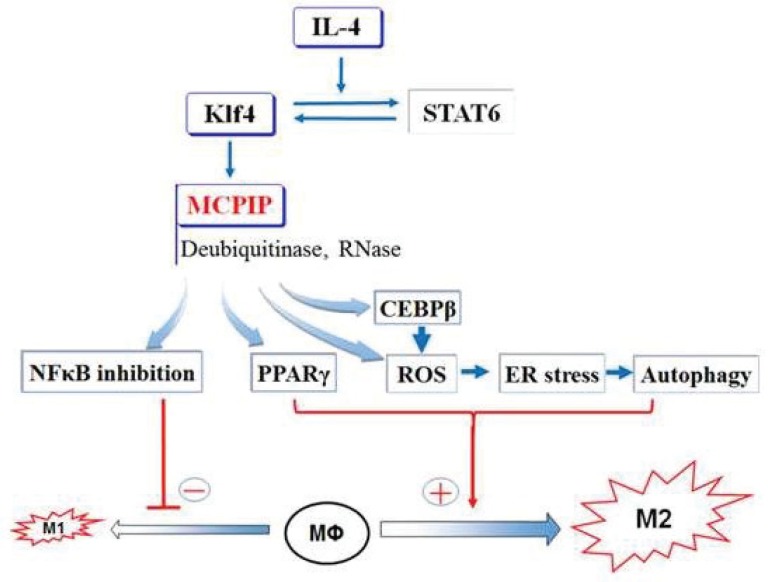
Scheme: Role of MCPIP in M2 polarization

Il-4 binds to its receptor IL-4Ra to cause phosphorylation of STAT6 to activate the JAK-STAT6 pathway to cause transcriptional activation of genes such as PPARγ. PPARγ regulates fatty acid metabolism promoting aerobic respiration in M2 macrophages. Many of the M2 markers in the mouse are induced by STAT6 including Arg1, Mrc1, Fizz1 and Ym1. It has been demonstrated that STAT6 and KLF4 induce each other and function cooperatively to induce M2 polarization [4]. How the transcription factors STAT6/KLF4 induce the biological processes that are necessary for IL-4- induced M2 polarization was only recently elucidated. A protein that plays a central role in this M2 polarization was identified as the first member of a novel class of zinc finger proteins induced by MCP-1 treatment of human peripheral blood monocytes with MCP-1 and thus, named MCPIP. Since then it has been demonstrated that many inflammatory agents produced by M1 macrophages induce MCPIP. The promoter of the gene that encodes MCPIP contains multiple KLF4 binding sites. It was recently demonstrated that IL-4 treatment of murine macrophages induces not only STAT6 and KLF4 but also MCPIP as well as M2 markers [5]. That the M2 polarization induced via STAT6/KLF4 is mediated through MCPIP induction has been demonstrated. That MCPIP mediates M2 polarization in vivo was demonstrated by the finding that macrophages from mice with myeloid cell-specific knockout of MCPIP are incapable of IL-4 induced M2 polarization. Conversely, macrophages from mice with myeloid cellspecific overexpression of MCPIP supressed expression of M1 markers and elevated expression of M2 markers IL- 4-induced M2 polarization is accompanied by inhibition of M1 polarization mediated via inhibition of NFkB activation and KLF was reported to be involved in this inhibition. This KLF4 inhibition of M1 polarization was demonstrated to be mediated via MCPIP. Transcription factor C/EBPβ and PPARγ that are known to promote M2 polarization were recently shown to be induced by IL-4 via MCPIP demonstrating the central role of MCPIP in M2 polarization (Figure 1).

It has been reported in recent years that IL-4 induction of M2 polarization involves ROS production, ER stress, and autophagy. We demonstrated that IL-4 causes sequential induction of ROS production, ER stress and autophagy leading to expressions of M2 markers in macrophages via MCPIP, again demonstrating the central role of MCPIP in IL-induced M2 polarization [5].

In M2 polarization the biological functions of STAT6 and KLF4 are mediated via MCPIP that has deubiquitinase and RNase activities. With mutants that have only one of these activities it was demonstrated that both catalytic activities of MCPIP are required for mediating the IL-4 induced M2 polarization. Specific roles of the catalytic activities of MCPIP in inducing the biological processes involved in M2 polarization remain to be elucidated. RNase activity of MCPIP is known to cause degradation of mRNA that encodes inflammatory cytokines and their receptors thus inhibiting M1 polarization. Anti-Dicer RNase activity that suppress miR production is likely to be important in the role of MCPIP in macrophage polarization. In fact, it was found that expression of M2 associated miRs 223 and 146 was enhanced by MCPIP but this induction was suppressed by loss of RNase activity of MCPIP. Production of M1- associated miRs 155 and 125 was strongly suppressed by MCPIP when compared to its RNase dead mutant. These results give a glimpse at a possible role of the RNase activity of MCPIPin M2 polarization. The presence of large number of miRs and the fact that each miR has large number of potential targets make the elucidation of the functions of the anti-Dicer RNase activity of MCPIP very complex and difficult.

Evidence indicates that deubiquitinase activity of MCPIP probably involves inhibition of NFkB activation by removal ubiquitin from components like TRAF6 and high molecular weight polyubiquitin involved in IKB kinase activation required for NFkB activation [5]. Little is known about the ubiquitome involved in macrophage polarization. Even though the extensively studied deubiquitinases CYLD and A20 are also known to negatively regulate NFkB activation, as does MCPIP, the target substrate and physiological functions of virtually all deubiquitinases remain obscure. Determination of the functions of the two catalytic activities of MCPIP is further complicated by the fact that deubiquitinase activity can affect miR production and function just as miRs can affect ubiquitination and deubiquitination of components that play critical roles in macrophage polarization and other related processes such as angiogenesis, adipogenesis and osteoclasogenesis

Macrophage polarization plays critical roles in many human inflammatory diseases including would healing, atherosclerosis, obesity and insulin resistance, cancer, rheumatoid arthritis, bacterial and parasitic infection [3, 6]. Understanding the mechanisms by which MCPIP regulate macrophage polarization might help in devising ways to modulate polarization for clinical benefits. Since deubiquitinase and RNase activities are involved in modulating different aspects of polarization, differential interference in the two activities might provide means to fine tune the MCPIP functions for clinical use. These aspects have yet to be experimentally explored.
